# Neurological Manifestations of an Old Disease: A Case Report

**Published:** 2013

**Authors:** Zia ISLAMI, Mohammad Hosein ATAEE NAKHAEI

**Affiliations:** 1 Department of Pediatrics, Shahid Sadoughi University of Medical Sciences, Yazd, Iran

**Keywords:** Neonatal tetanus, Poor feeding, Neonatal seizure

## Abstract

**Objective:**

Neurological manifestations of neonatal disorders have various causes, among them neonatal tetanus, albeit rare, is a potentially fatal and preventable disease, which is seen in underdeveloped and developing countries. Although the disease has been eradicated from I.R. Iran, pregnant women immigrating to Iran from neighboring countries, especially from eastern border, may carry a risk of neonatal tetanus to the child due to inadequate tetanus immunization and inappropriate post-delivery care. It is then important to maintain a high index of suspicion for early diagnosis and prompt treatment, when infants present with poor feeding and abnormal behavior.

Case presentation Here, we report the clinical course of a newborn with neonatal tetanus, who was admitted with complaints of poor feeding and muscle rigidity, more than a decade after eradication of the disorder.

## Introduction

Tetanus is an acute, spastic paralytic disease caused by the tetanus toxin which is released from Clostridium tetani, a naturally inhabitant of dust, soil, clothing, skin, and the gastrointestinal tracts of animals and human. Clinically, there are four types of tetanus: generalized, local, cephalic, and neonatal. Neonatal (or umbilical) tetanus, also called tetanus neonatorum, the most common form, is very rare in developed countries, but has not been eradicated in developing and underdeveloped countries and is a major cause of infant mortality and morbidity in these regions. The disorder is almost always lethal, thus early diagnosis and prompt interventions are crucial. The only effective preventive strategy against this infection is maternal vaccination.

## Case report

**Fig 1:**
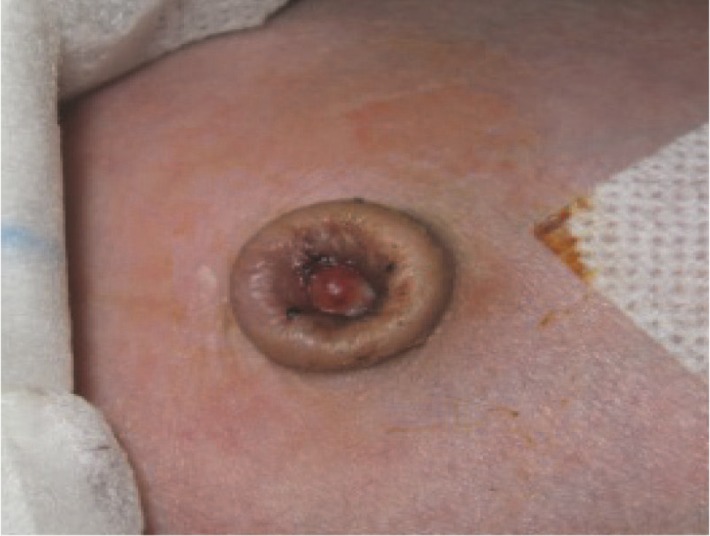
Umbilical cord stump showed malodorous discharge and erythema

On April 24th, 2011, an eight-day-old newborn girl, without any previous medical problems was taken to Shahid Sadoughi hospital of Yazd province of Iran by her parents who reported a 24-hour history of poor feeding, lock-jaw, cyanosis, and spasticity of limbs coupled with frequent tonic-clonic movements from about 10 hours before. On admission, the newborn had central cyanosis, trismus, increased general muscle tone, and hyperresponsiveness to external audio-visual stimuli. The cardiopulmonary examination was normal. The umbilical cord had been covered with dirty crust, with a foul-smelling yellowish-green discharge beneath it (Fig. 1)

Her core temperature was normal, and laboratory investigations, including complete blood count, blood chemistry, urinalysis, and cerebrospinal fluid examination were not remarkable. Neonatal tetanus, then, was diagnosed based on the clinical characteristics. The newborn was fully monitored in a private room to reduce external stimuli.

**Fig 2:**
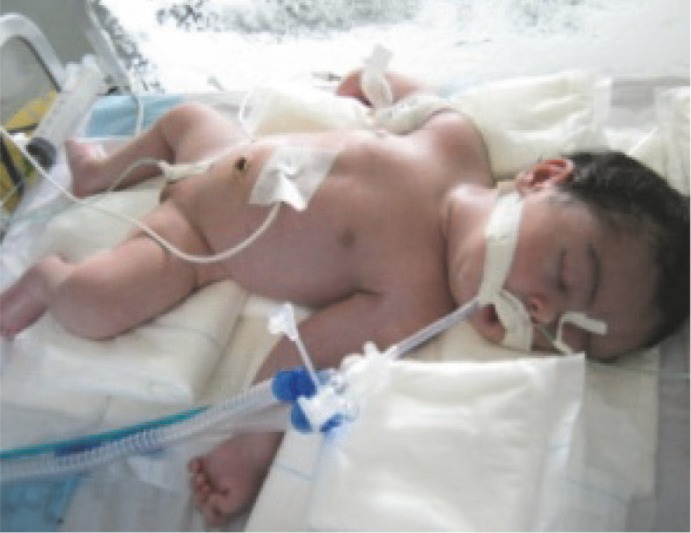
The baby, after seven day, underwent mechanical ventilation due to sudden respiratory arrest

She was treated with human tetanus immunoglobulin, cefepime, metronidazole and vancomycin, and was vaccinated with diphtheria-pertussis-tetanus (DPT), when discharged. Her spasticity was controlled by diazepam. However, because of persistent spasticity, phenobarbital and chlorpromazine were added, and then the patient become seizure-free and the treatment continued for 40 days. After seven days, she began to suffer from prolonged apnea due to pneumonia and needed mechanical ventilation for 15 days (Fig. 2). She was discharged after 55 days on June 18th, with no clear sequelae. Her ophthalmologic, cardiovascular, and neurological examinations were normal, except for mild brain atrophy in CT scan. Her re-examination after six weeks revealed a healthy baby without any neurological abnormalities.

The mother, a young multipara Afghan woman, born in her motherland, but become pregnant in Iran, had never been immunized. She had no complications during her pregnancy, with no prenatal medical care. The newborn was delivered at home with the aid of an unqualified midwife in unsanitary conditions. Here, the midwife cut the umbilical cord with an used razor and tied it with a non-sterile string.

## Discussion

Neonatal tetanus, with report of 180,000 newborn deaths in 2002, results from umbilical stump contamination during unsanitary delivery, coupled with inappropriate maternal immunization. It typically manifests at the end of the first week, usually during 3-12 days of birth, as excessive irritability and difficulty in sucking and swallowing (1-3). The source of the infection may be the contaminated hands of the birth attendant and/or more frequently, the dressing of the cord stump (4). Neonatal tetanus should be one of the differential diagnosis of the newborns presenting with poor feeding, seizures, stiffness, cyanosis and weak cry (Tablet 1) (4). Tetanus management involves the neutralization of toxins using tetanus immunoglobulin, administration of antibiotics to stop toxin production, as well as intensive support for the control of muscular spasticity and sufficient maintenance of respiratory tract (5). Tetanus is completely preventable by active immunization of all women of reproductive age (5, 6). Officially speaking; neonatal tetanus has reached elimination phase in I.R. Iran due to widespread maternal immunization and hygienic delivery practices (7, 8).

In recent decades, I.R. Iran had been flooded with mass immigration from nearby countries (Afghanistan, Pakistan, and Iraq, especially due to long standing war and poor socio-economic situation), which caused a serious problem for health care system of I.R. Iran. Here, many risk factors, including contaminated razor and birthing bed, unhealthy cord care, and inappropriate hand washing resulted in neonatal infection. This case has documented a clear indication of immunization and clean delivery for all the immigrant women to better control of the neonatal tetanus in the region.

In 1989, the 42nd World Health Assembly called for elimination of neonatal tetanus (9). Although maternal and neonatal tetanus elimination strategies continues to progress, by December 2010, 39 countries have not reached this goal (10). It is hoped that this goal will be achieved in the near future. In addition, Mothers’ education in favor of promotion of tetanus vaccination and hygienic childbearing practices is necessary for tetanus prevention.

**Table 1:** Differential Diagnosis of Neonatal Tetanus

Tetany (Hypocalcaemia, Hypomagnesemia)
Neonatal seizures
Abnormal movements(Chorea, Myoclonus)
Intracranial hemorrhage
Drug withdrawal
Infections (Sepsis, Meningitis, Encephalitis)
Kernicterus
Inborn errors of metabolism

**In conclusion,** although neonatal tetanus is a rare disorder in I.R. Iran, however, it is important to maintain a high index of suspicion for early diagnosis and prompt treatment, when infants present with poor feeding and seizure-like symptoms.
